# Gd-EOB-DTPA-Enhanced MRI for Detection of Liver Metastases from Colorectal Cancer: A Surgeon's Perspective!

**DOI:** 10.1155/2013/572307

**Published:** 2013-04-09

**Authors:** Kelly J. Lafaro, Panayota Roumanis, Aram N. Demirjian, Chandana Lall, David K. Imagawa

**Affiliations:** University of California, Irvine, 333 The City Boulevard West, Suite 1205, Orange, CA 92868, USA

## Abstract

Colorectal cancer affects over one million people worldwide annually, with the liver being the most common site of metastatic spread. Adequate resection of hepatic metastases is the only chance for a cure in a subset of patients, and five-year survival increases to 35% with complete resection. Traditionally, computed tomographic imaging (CT) was utilized for staging and to evaluate metastases in the liver. Recently, the introduction of hepatobiliary contrast-enhanced magnetic resonance imaging (MRI) agents including gadolinium ethoxybenzyl diethylenetriamine pentaacetic acid (Eovist in the United States, Primovist in Europe, or Gd-EOB-DTPA) has proved to be a sensitive method for detection of hepatic metastases. Accurate detection of liver metastases is critical for staging of colorectal cancer as well as preoperative planning.

## 1. Introduction

Colorectal cancer is one of the most common cancers worldwide with nearly one million people diagnosed each year. The liver is the most common site of distant metastases from colorectal cancer. Up to 70% of all patients with colorectal cancer will develop hepatic metastases at some point in their lifetime, and one-third of these will have metastases confined only to the liver [1, 2]. 

In metastases from colorectal adenocarcinoma, locoregional therapies are available including radiofrequency ablation and resection, which provide a survival benefit for patients with limited disease. Hepatectomy for liver metastases from colorectal cancer is the gold standard of treatment and provides the only chance for cure. Complete resection of all disease has been associated with a five-year survival ranging between 22% and 58% [3]. However, incomplete resection does not increase patient survival [4, 5]. Median survival for patients with untreated but potentially resectable metastases is 8 months, with a 5-year survival of less than 5% [2]. The paradigm for resection of colorectal metastases has changed from excluding patients with more than 3-4 liver metastases, periportal lymphadenopathy, or metastases within 1 cm of major vessels to only excluding those in which a margin-free resection cannot be achieved without preserving at least a 20% future liver remnant, or 30% if the patient has undergone chemotherapy [6–8]. Identification and resection of liver lesions often rely on high-quality cross-sectional imaging studies, and these images are an indispensable tool in the treatment planning process [9].

 Historically, computed tomography (CT) has been used to stage and evaluate the liver in patients with colorectal adenocarcinoma. However, the introduction of magnetic resonance imaging (MRI) has proven to be a highly effective way to evaluate liver parenchyma [10–12]. In 1997, the introduction of specific hepatobiliary contrast agents for MRI further enhanced and strengthened this imaging modality. In addition to a more sensitive way to image metastatic lesions, the introduction of MRI allowed radiologists to avoid the risk of contrast-induced nephropathy in patients with eGFR <40 mL/min in healthy patients and <60 mL/min in diabetic patients [13].

Early publications evaluating the use of gadolinium-based contrast agents showed an advantage of MRI over CT [14]. Recently, multiple studies have demonstrated the superiority of Gd-EOB-DTPA-enhanced MR over CT for detection of liver metastases [15].

## 2. Hepatobiliary Contrast Agents

Mangafodipir trisodium (MT) was the first specific hepatobiliary contrast agent. It was introduced in 1997 and approved as “an adjunct to MRI in patients to enhance the T1w images used in the detection, localization, characterization, and evaluation of lesions in the liver” (package insert, [16]).  MT is a manganese-based agent, which shows hepatic enhancement as well as some biliary contrast. The agent had limited assessment of vascular structures due to its inability to be administered as a bolus and the agent was taken off the market in the United States in 2005 due to concerns regarding toxicity [16, 17].

Gadobenate dimeglumine (MultiHance, Gd-BOPTA) was approved by the FDA in 2004 for use as an MRI contrast agent. Gd-BOPTA acts as both an extracellular agent as well as a hepatobiliary contrast agent. While it is approved for hepatobiliary imaging in Europe, it is used off-label in the United States. 3% to 5% is taken up by hepatocytes and excreted into the biliary system which allows for its hepatobiliary specificity [18, 19].

 In 2008, gadoxetic acid (Gd-EOB-DTPA, Eovist, Primovist) was FDA approved in the United States for the detection and characterization of liver lesions. It had previously been approved in Europe in 2004 as Primovist. Eovist is a gadolinium-based contrast agent with approximately 50% uptake into hepatocytes and subsequent biliary excretion [9]. After administration, it is distributed into the vascular and extravascular spaces allowing for arterial, portal venous, and late dynamic phases. This is similar to nonspecific extracellular gadolinium contrasts; however, it adds information during the hepatobiliary phases [20–23]. It offers strong, early intravascular contrast allowing for dynamic phase imaging facilitated by 11% protein binding. This leads to increased relaxivity, thereby leading to increased signal enhancement in the blood and liver [24]. Due to its high relaxivity, dosing is also much lower than with other gadolinium compounds. Gd-EOB-DTPA is approved at a dose of 0.1 mL/kg to 0.025 mmol/kg body weight, This is one-fourth the dose of other gadolinium agents used in liver MRI. It is thought to be absorbed into hepatocytes by the canalicular multispecific organic anion transporter 8 (OATP8) with subsequent excretion into the bile via multidrug resistant protein 3 (MRP3) [25]. The hepatobiliary phase contrast enhancement peaks at 20 minutes and persists for more than 2 hours [26]. Gd-EOB-DTPA demonstrates earlier onset, as well as longer duration of contrast than Gd-BOPTA, which facilitates imaging and image quality [27]. 

Gd-EOB-DTPA's elimination half-life in healthy patients is roughly 55 to 57 minutes. Its elimination pathway is unique compared to other gadolinium agents. It is eliminated equally (50%) through the renal and hepatobiliary systems. In patients with one impaired pathway, the other elimination pathway will remove a larger percentage of the dose. For this reason, patients with renal or hepatic impairment do not require dose adjustment [24]. It does carry the black box warning given to all gadolinium-based contrast agents because of its association with nephrogenic fibrosing dermopathy. This is a rare occurrence and tends to affect those with end-stage renal disease already on dialysis, and therefore it is recommended to avoid the use of Gd-EOB-DPTA in patients with an eGFR <25 [28]. The most frequent adverse reactions include nausea, vasodilation, headache, taste perversion, and injection site pain [29]. Eleven patients out of 162 (6.8%) in a phase III trial reported a total of 21 adverse events. Of these, one was determined to be definitely related to GD-EOB-DPTA administration, 5 probably related, seven possibly related, one unlikely to be related, and seven not related. Eovist-enhanced MRI is invaluable in patients with severe allergies to iodinated contrast agents, such as prior anaphylaxis, who should not receive Iodine during CT imaging. Gadolinium-based agents tend to be fairly safe in this subset of patients.

## 3. Characterization of Liver Lesions with MRI

The use of dynamic contrast-enhanced MRI with gadolinium-based contrast agents for the characterization of benign and malignant liver lesions has been well described in a growing body of literature [30, 31]. Liver lesions with minimal or no hepatocyte function do not show accumulation of Eovist. These lesions include cysts, hemangiomas, metastases, the majority of hepatocellular carcinomas, and hepatic adenomas. In these cases, liver lesions will be more easily detected secondary to increased contrast between the lesions and the normal enhancing hepatocytes in the liver. On the other hand, hepatic lesions with functioning hepatocytes—including focal nodular hyperplasia and a small percentage of well-differentiated hepatocellular carcinomas—generally take up Eovist during the hepatocyte phase. These lesions will be enhanced during the hepatocyte phase and demonstrate a distinct pattern of enhancement. From this, a variety of algorithms to characterize liver lesions based on enhancement patterns have been developed. However, when interpreting images acquired with gadolinium-based contrast agents, understanding the pathophysiology and pharmacokinetics is important for image interpretation. 

Gd-EOB-DTPA has unusual pharmacokinetics in that it is the only gadolinium agent which is taken up in equal amounts by the extracellular space and functioning hepatocytes, demonstrating a biphasic action. The dynamic phase of gadoxetic acid occurs during the first 2 minutes after injection. During this phase, normal hepatocytes as well as lesions containing hepatocytes exhibit contrast enhancement due to hepatocellular uptake. The enhancement pattern depends on vascular supply, the presence of functioning hepatocytes, and characteristics of the biliary structures within the lesion. The normal hepatocytes then slowly excrete the contrast agent into the biliary tree, which is referred to as the “hepatobiliary phase.” For gadoxetic acid, the duration of this phase is 20–120 minutes where hepatocytes have excreted the contrast into the bile and have reached a level optimal for interpreting images of both the biliary system and hepatic lesions [26, 27, 32].

## 4. Metastatic Disease

Metastatic lesions are typically present on gadolinium-based contrast-enhanced imaging with peripheral rim enhancement and lack of central enhancement in the dynamic phase when central tumor necrosis is present. During the hepatobiliary phase, contrast uptake by hepatocytes provides contrast between the liver parenchyma and metastatic disease causing metastases to appear hypointense. In addition, during the hepatobiliary phase, rim enhancement [33] and a “target sign” [34] have been described in metastases. Initially, as mentioned previously, gadopentetate dimeglumine was the only gadolinium-based contrast available. The introduction of Gd-EOB-DPTA allowed for increased uptake by hepatocytes, and Vogl et al. (1996) demonstrated lesion to liver contrast superiority of Gd-EOB-DPTA over gadopentetate dimeglumine, and a statistically significant improvement in detection rate in metastases, hepatocellular carcinoma, and hemangiomas [35]. 

Initial studies using gadolinium-based contrast agents showed advantages of MR over CT, but with some mixed results [14]. Since the initial studies, multiple groups have evaluated Gd-EOB-DPTA-enhanced MRI for detecting liver metastases. 

In 2004, Kim et al. demonstrated improved tumor to liver contrast using gadobenate dimeglumine in the hepatobiliary phase, leading to detection of more metastases compared to dynamic imaging alone [34]. In their paper, they describe the appearance of a “target sign” with central hyperenhancement and a hypointense rim [34].

The European EOB Study Group (Huppertz et al. 2004) looked at 302 lesions in 13 patients with biopsy or intraoperative ultrasound proven focal liver lesions. 81 of these patients had metastases from a colorectal tumor primary. T1 and T2 phase MRIs, pre- and post-Gd-EOB-DTPA, were performed and evaluated in a blinded fashion by three radiologists. In 21 of the 129 patients, results differed between pre- and postcontrast MRI, and 19 of these were correct in the Gd-EOB-DTPA group, resulting in a significant (*P* < 0.001) difference in the correct detection with Gd-EOB-DTPA. It also showed a 7% increase in correct lesion classification with Gd-EOB-DTPA as compared to precontrast MRI [36].

Bluemke et al. (2005) showed that the percentage of lesions that were correctly classified as malignant or benign was 2–15% greater for blinded readers comparing MR images with Gd-EOB-DTPA with helical CT images of the same patients, with an increase in false positive lesions identified using CT. False positive results in liver imaging for metastases from colorectal cancer can impact surgical planning, delay excision of the primary tumor, and result in unnecessary surgery [37]. 

**Figure 1:**
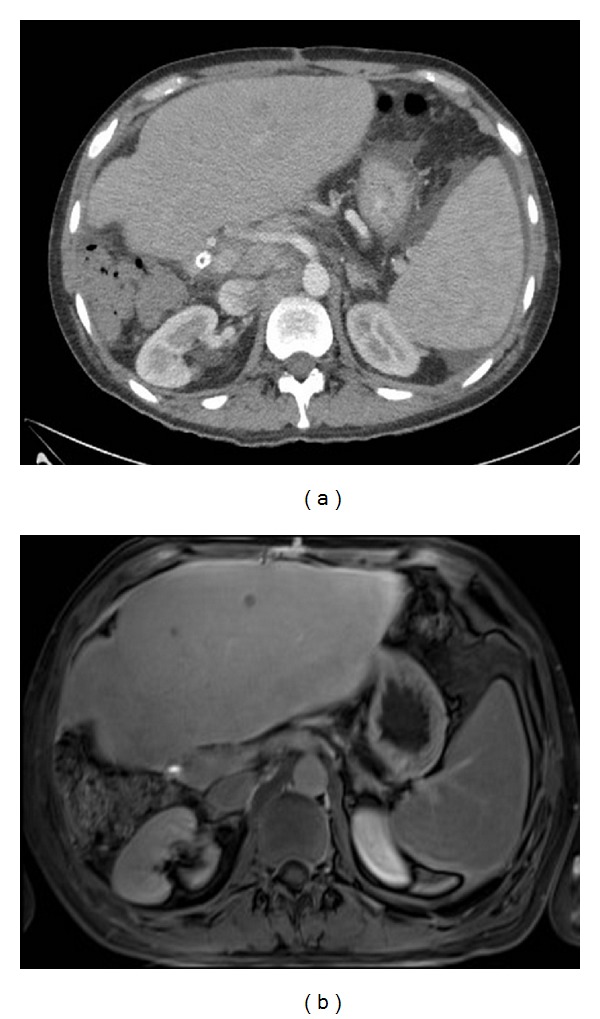
Contrast CT compared with Gd-EOB-DTPA-enhanced MRI. 65 y. male after right hepatic lobe resection. Routine follow-up CT scan (a) shows subtle low attenuation lesions in the left hepatic lobe, clearly seen on MRI with Eovist (b).

**Figure 2:**
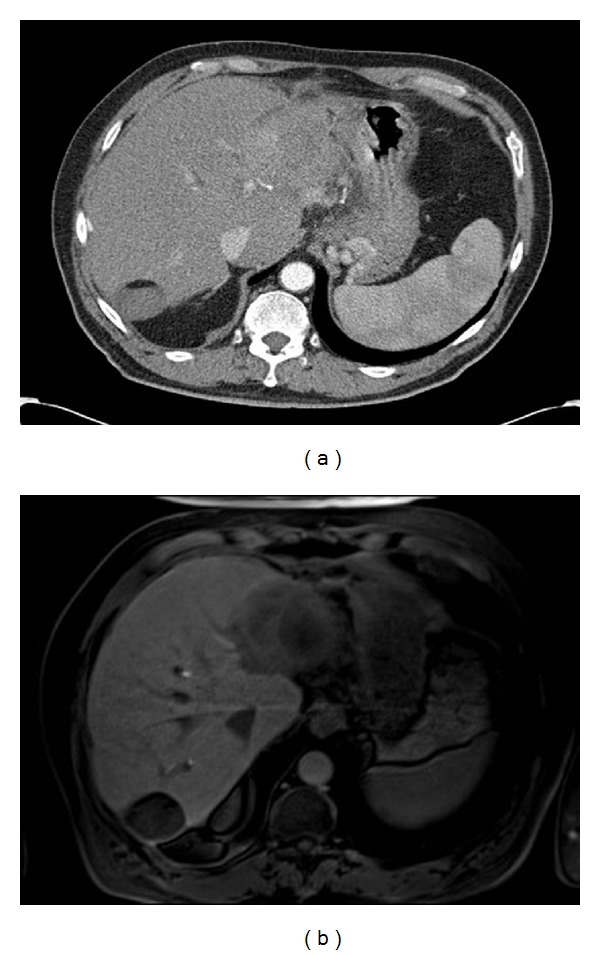
Contrast-enhanced CT compared to Gd-EOB-DTPA-enhanced MRI in the same patient. 69 y. male with colorectal cancer after RFA of segment 6 lesion. New CT and MRI with Eovist ordered for elevated CEA. CT (a) shows a mildly heterogeneous area in segment 2. MRI with Eovist (b) shows a clearly demarcated lesion measuring over 7 cm consistent with metastasis.

In 2006, Halavaara et al. showed superiority of Gd-EOB-DPTA compared to CT and diffusion weighted MR. They found increased lesion identification (95% versus 89%), sensitivity (95 versus 92%), and specificity (94 versus 90%) with MRI compared to CT [38]. This was further supported by Hammerstingl et al. (2008) who looked at 302 lesions and showed that the frequency of correctly detected lesions was significantly higher (10.44%) on Gd-EOB-DPTA-enhanced MRI compared with biphasic helical CT scan using histopathology or intraoperative ultrasound as confirmation. This superiority held true when looking at lesions with a diameter <1 cm. Interestingly, a change in surgical therapy was documented in 19 of 131 patients (14.5%) after Gd-EOB-DTPA-enhanced MRI [29]. This superiority of Gd-EOB-DTPA to CT scan has been witnessed in multiple studies, as well as at our own institution. Contrast of metastases from colorectal cancer on CT imaging versus Gd-EOB-DTPA-enhanced MRI is clearly demonstrated in Figures 1 and 2. 

Studies by Ichikawa et al. (2010) have demonstrated superiority of gadoxetic-acid-enhanced MR over unenhanced MR and triphasic contrast-enhanced spiral CT for detection of metastases with regard to sensitivity. This was true for lesions <20 mm in diameter; however, the lesions <20 mm were not broken down by lesion type [15].

In the same year, Shimada et al. looked at 45 patients undergoing abdominal MRI. A total of 51 hepatic metastases were examined by two independent observers. 7 of these lesions were seen on Gd-EOB-DTPA but were missed on DWI by both observers. 2 metastases very close in proximity to hepatic vessels were difficult to detect on Gd-EOB-DTPA but were seen clearly on DWI. In this study, Gd-EOB-DTPA-enhanced MRI showed statistically significant higher accuracy in detection of small lesions (<2 cm) than DWI. However, only slightly more than half of these metastases were confirmed histologically; the remainder were considered to be metastases on the basis of tumor growth on follow-up radiologic examinations [39].

**Figure 3:**
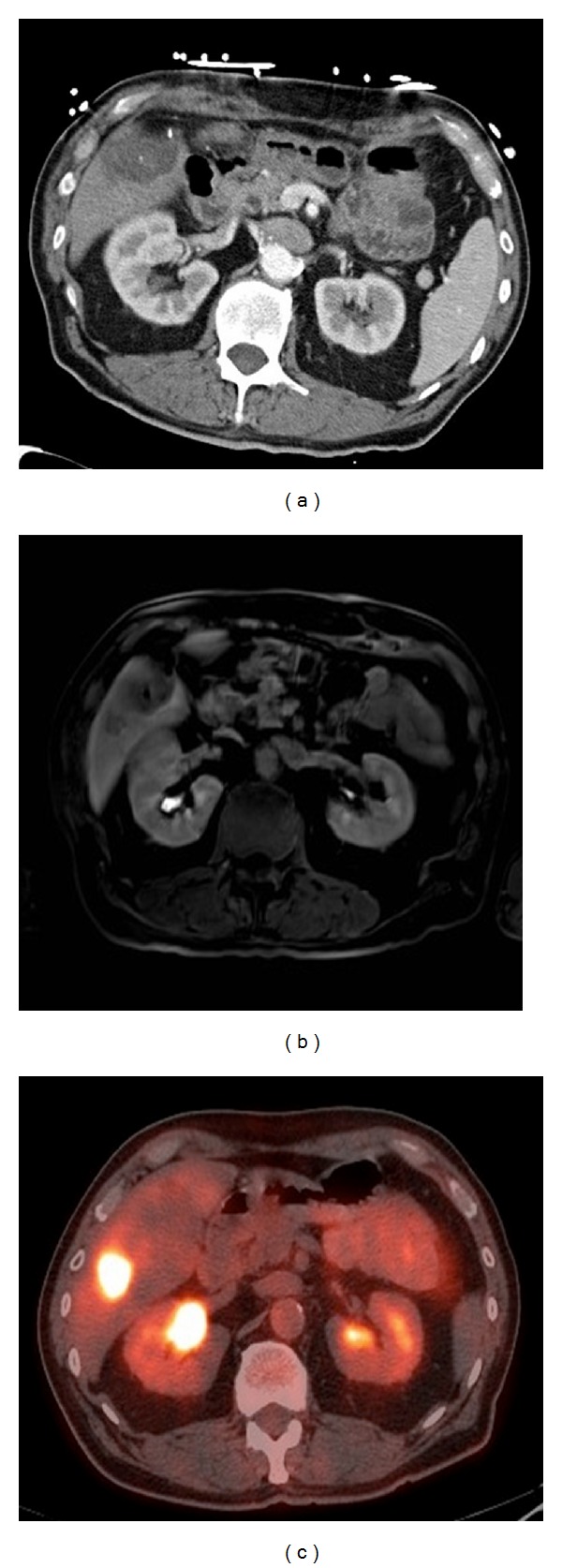
Liver metastases in the same patient compared with contrast-enhanced CT, Gd-EOB-DTPA-enhanced MRI and PET/CT scan. 76 y. male with history of rectal cancer after neoadjuvant chemotherapy and radiation followed by low anterior resection. Segment 5 and caudate lesions, now with segment 6 lesion not seen on CT scan (a) but present on PET (c) and MRI with Eovist (b).

Two studies have compared Gd-EOB-DTPA-enhanced MRI with PET/CT. Donati et al. (2010) looked at 85 liver lesions in 29 patients. 45 of these were metastases from a colorectal primary. The metastases were not divided out by primary cancer type [40]. When looking at the lesions as a whole, there was a significant difference in lesion detection between PET/CT and Gd-EOB-DTPA MRI (64% and 85% resp., *P* = 0.002). There was also a significant difference in detection (29% and 71% *P* = 0.013) for lesions less than 1 cm in diameter. This study is very limited by the fact that it did not differentiate between benign and malignant lesions [40]. Following this study, Seo et al. (2011) compared the diagnostic accuracy of Gd-EOB-DTPA MRI to 18F-flourodeoxyglucose positron emission tomography/computed tomography (CE-PET/CT) for the detection of liver metastases, specifically from colorectal cancer. This study retrospectively looked at 135 metastases from 68 patients who underwent both imaging studies and were reviewed by 2 radiologists independently. They found significantly higher diagnostic accuracy and sensitivity of EOB-MRI than CE-PET/CT (*P* < 0.001). On subset analysis, 25 small lesions less than 1 cm in diameter were detected only with EOB-MRI [41]. This difference in lesion detection between PET/CT and Gd-EOB-DTPA-enhanced MRI is clearly demonstrated from a patient at our own institution as seen in Figure 3. 

Löwenthal et al. (2011) demonstrated the superiority of Gd-EOB-DTA-enhanced MR in the hepatobiliary phase to MR-DWI and MR for the detection of focal liver lesions when looking at 332 lesions (94.4%/100%, 78.3%/97.5%, and 81.5%/89.9% resp.). However, unlike Hammerstingl et al., this study did note that sensitivity for lesions <1 cm was higher for MR-DWI than for MR hepatobiliary phase images (0.98 versus 0.92) [42].

Chung et al. (2011) looked at a series of 47 patients with a total of 78 confirmed colorectal metastases comparing DWI and Gd-EOB-DTPA-enhanced MRI. In this study, regardless of lesion size (greater or less than 2 cm in diameter), significantly more lesions were detected when looking at both DWI and Gd-EOB-DTPA-enhanced images than with DWI imaging alone [43]. All lesions in this study were confirmed as metastases histopathologically. Interestingly, in this study, positive predictive value was higher in the DWI group compared to Gd-EOB-DTPA or the combined DWI and Gd-EOB-DTPA. 

Muhi et al. (2011) broadened the comparison and looked at the diagnostic accuracy of contrast-enhanced CT (CE-CT), contrast-enhanced ultrasound (CE-US), and superparamagnetic iron oxide-enhanced MRI (SPIO-MRI) for detecting colorectal hepatic metastases. 112 metastases in 46 patients were evaluated. For all lesions combined, sensitivity and area under the receiver operating characteristic curve of Gd-EOB-DTPA-enhanced MRI were significantly greater (95%) than CE-CT (63%) and CE-US (73%). For lesions less than 1 cm in diameter, sensitivity of Gd-EOB-DTPA-enhanced MRI was significantly greater than for CE-CT and CE-US. However, they did not find a significant difference in positive predictive value between any of the imaging modalities [44].

Most recently, Chen et al. performed a meta-analysis of 1900 lesions from 13 studies showing the sensitivity of Gd-EOB-DTPA-enhanced MRI for detection of liver metastases to be 93% and specificity 95% with statistically significant decreased sensitivities with lesions less than 10 mm in size (*P* = 0.001) [45]. 

The issue of imaging patients following neo-adjuvant chemotherapy who have developed hepatic steatosis was evaluated by Berger-Kulemann et al. (2012). In this study, 68 metastases were evaluated with triphasic MDCT and Gd-EOB-DTPA-enhanced MRI. All patients underwent surgical resection of liver metastases after evaluation. For lesions <1 cm diameter, MDCT detected only 41.9% while MRI detected 93% of metastases (*P* < 0.001). There was not a significant difference in lesions >1 cm in diameter between MDCT and Gd-EOB-DPTA enhanced MRI (97% and 100% resp.) [46].

While there have been multiple studies looking at the sensitivities and specificities of Gd-EOB-DPTA contrast enhanced MRI compared to other imaging techniques, economic considerations have become an increasingly important aspect of patient care in the United States. Zech et al. performed a cost analysis comparing MRI with Eovist, extracellular enhanced MRI, and three-phase MDCT as the initial evaluation of patients with metachronous colorectal liver metastases in Germany, Italy, and Sweden. It demonstrated that MRI with Eovist required fewer additional imaging studies (8.6%) than extracelluar-enhanced MRI (18.5%) and MDCT (23.5%). While MRI with Eovist has the highest initial imaging cost of the three modalities studied, it was in fact cost saving when reimaging, and the cost of modified and unnecessary surgical procedures was factored into the equation [47].

## 5. Conclusions 

The ideal preoperative imaging study would provide diagnostic information which is highly sensitive and has a low rate of false positives. The studies described above have shown that hepatobiliary phase imaging with gadoxetic acid is safe and offers increased sensitivity in the detection of metastases due to the superior liver-lesion contrast generated by the avid uptake of gadolinium into the background of liver parenchyma. In addition, it has a lower rate of false positives than helical CT scan. These are important aspects of imaging in preoperative planning for resection of metastases from colorectal cancer. 

Accurately mapping the location and number of metastases from colorectal cancer is crucial for the surgeon's preoperative planning process. Not only can it dramatically alter an operation from a minor wedge resection to a much larger anatomic procedure, but also it allows the surgeon to counsel the patient more accurately regarding the procedure they will require to excise the metastases. This is supported by the studies discussed previously where operative plans changed after reviewing Gd-EOB-DPTA enhanced MRI imaging [29]. In addition, false positive results can lead to unnecessary procedures for patients, and in a time when “liver first” surgery is accepted and increasingly popular, it can unnecessarily delay the resection of the primary tumor. Eovist-enhanced MRI is superior to other imaging modalities in the detection, localization, delineation, and management of patients with liver metastases from colorectal cancer.
